# Supplementary material for: Ras Pathway Activation and MEKi Resistance Scores Predict the Efficiency of MEKi and SRCi Combination to Induce Apoptosis in Colorectal Cancer
Source: Cancers (Basel). 2022 Mar 11;14(6):1451. doi: 10.3390/cancers14061451 (PMC8945886; doi:10.3390/cancers14061451)
Supplement: Supplementary file 1 [file cancers-14-01451-s001.zip › cancers-1603560-supplementary.pdf]

Supplementary Materials

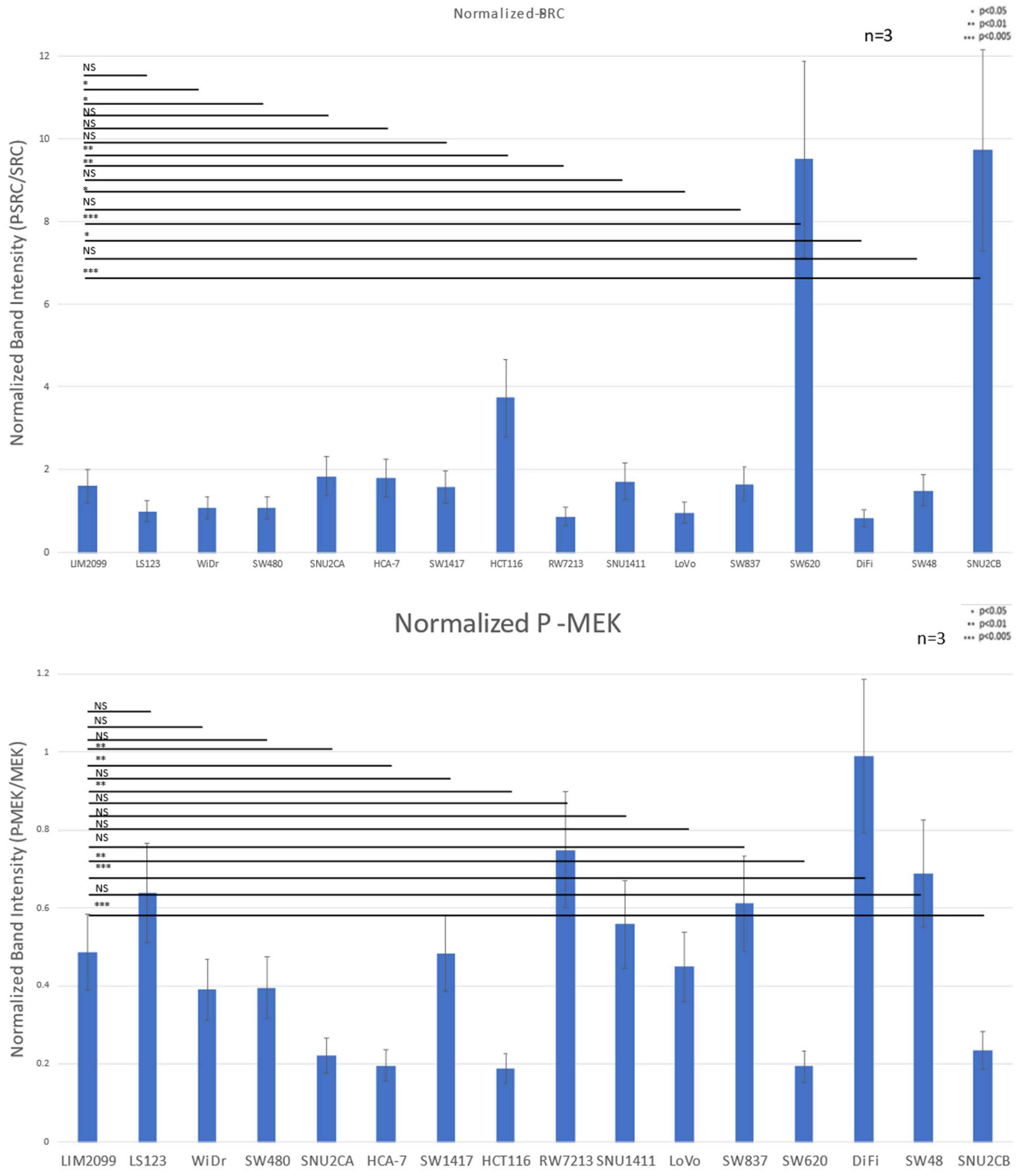

Figure S1. Band Density Analysis for western blots in Figure 1.

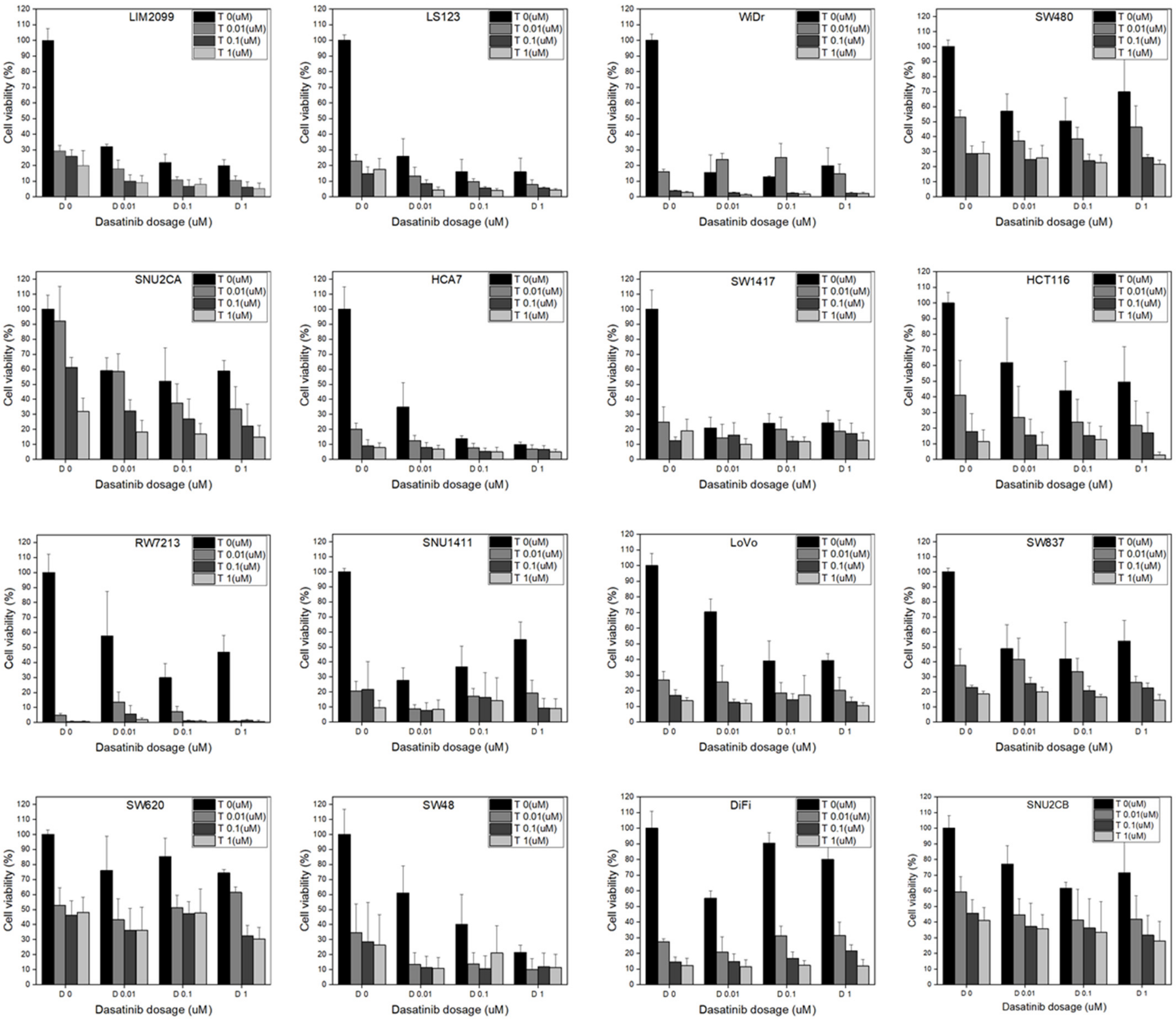

Figure S2. Dose dependent analysis of cell viability with Trametinib and/or Dasatinib for 16 CRC Cell lines.

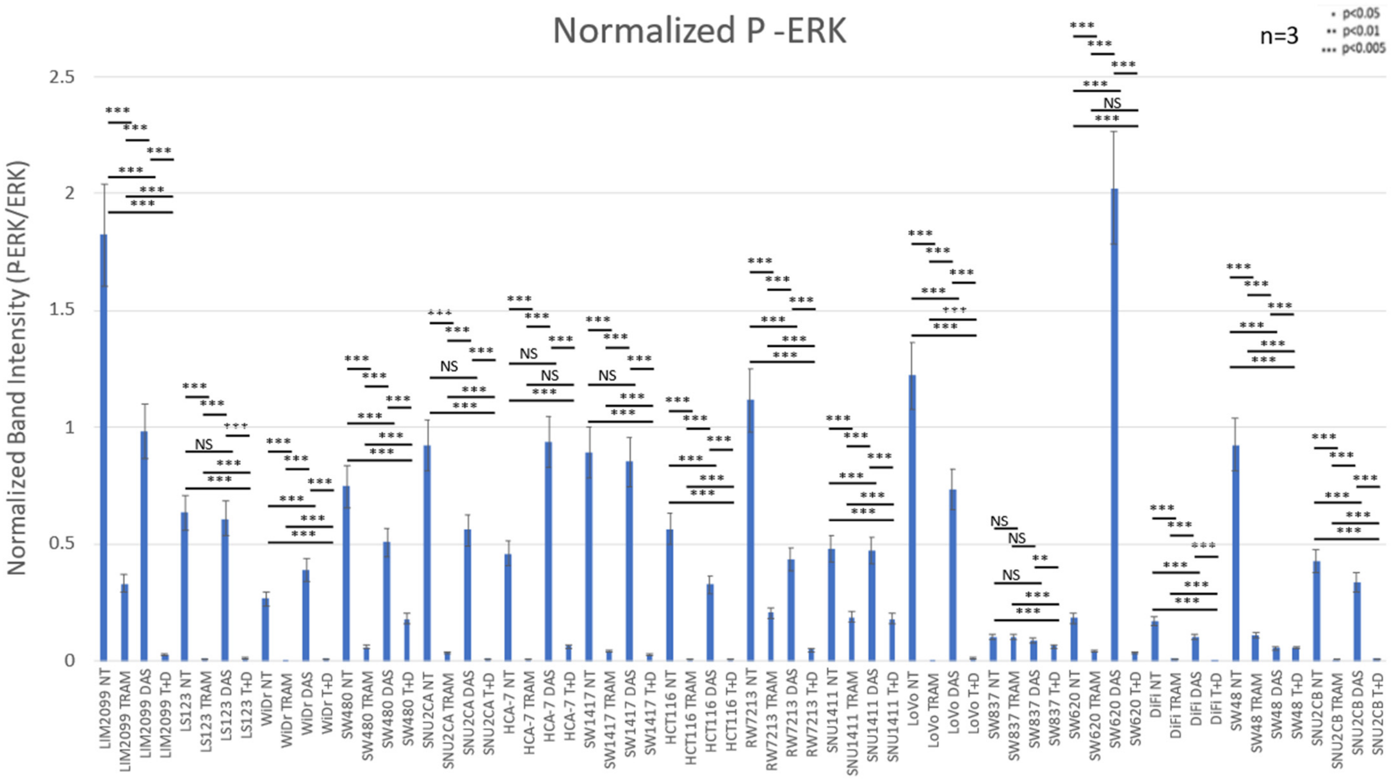
Normalized P -ERK
n=3
* p<0.05
** p<0.01
*** p<0.005
Normalized Band Intensity (P-ERK/ERK)
2.5
2
1.5
1
0.5
0
LIM2099 NT
LIM2099 TRAM
LIM2099 DAS
LIM2099 T+D
LS123 NT
LS123 TRAM
LS123 DAS
LS123 T+D
WiDr NT
WiDr TRAM
WiDr DAS
WiDr T+D
SW480 NT
SW480 TRAM
SW480 DAS
SW480 T+D
SNU2CA NT
SNU2CA TRAM
SNU2CA DAS
SNU2CA T+D
HCA-7 NT
HCA-7 TRAM
HCA-7 DAS
HCA-7 T+D
SW1417 NT
SW1417 TRAM
SW1417 DAS
SW1417 T+D
HCT116 NT
HCT116 TRAM
HCT116 DAS
HCT116 T+D
RW7213 NT
RW7213 TRAM
RW7213 DAS
RW7213 T+D
SNU1411 NT
SNU1411 TRAM
SNU1411 DAS
SNU1411 T+D
LoVo NT
LoVo TRAM
LoVo DAS
LoVo T+D
SW837 NT
SW837 TRAM
SW837 DAS
SW837 T+D
SW620 NT
SW620 TRAM
SW620 DAS
SW620 T+D
DiFi NT
DiFi TRAM
DiFi DAS
DiFi T+D
SW48 NT
SW48 TRAM
SW48 DAS
SW48 T+D
SNU2CB NT
SNU2CB TRAM
SNU2CB DAS
SNU2CB T+D

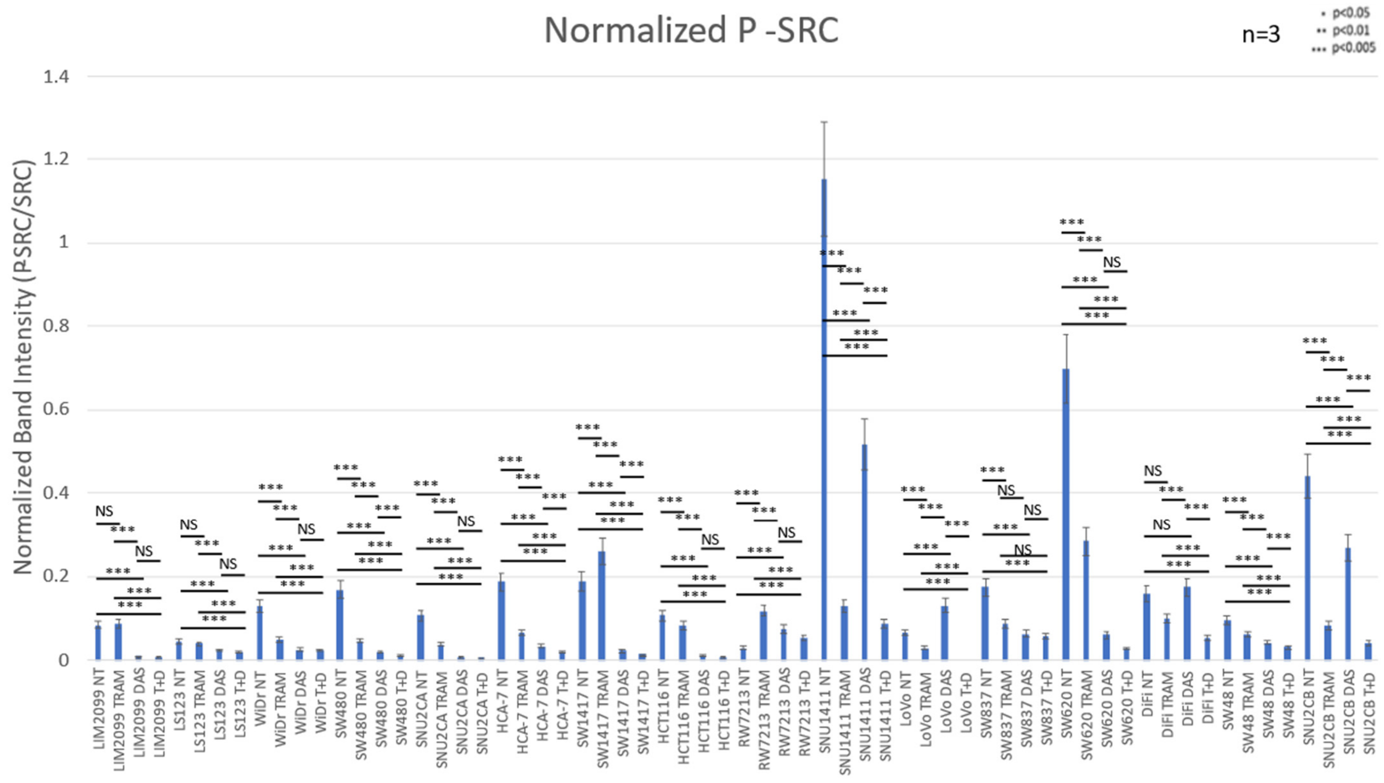
Normalized P -SRC
n=3
* p<0.05
** p<0.01
*** p<0.005
Normalized Band Intensity (P-SRC/SRC)
1.4
1.2
1
0.8
0.6
0.4
0.2
0
LIM2099 NT
LIM2099 TRAM
LIM2099 DAS
LIM2099 T+D
LS123 NT
LS123 TRAM
LS123 DAS
LS123 T+D
WiDr NT
WiDr TRAM
WiDr DAS
WiDr T+D
SW480 NT
SW480 TRAM
SW480 DAS
SW480 T+D
SNU2CA NT
SNU2CA TRAM
SNU2CA DAS
SNU2CA T+D
HCA-7 NT
HCA-7 TRAM
HCA-7 DAS
HCA-7 T+D
SW1417 NT
SW1417 TRAM
SW1417 DAS
SW1417 T+D
HCT116 NT
HCT116 TRAM
HCT116 DAS
HCT116 T+D
RW7213 NT
RW7213 TRAM
RW7213 DAS
RW7213 T+D
SNU1411 NT
SNU1411 TRAM
SNU1411 DAS
SNU1411 T+D
LoVo NT
LoVo TRAM
LoVo DAS
LoVo T+D
SW837 NT
SW837 TRAM
SW837 DAS
SW837 T+D
SW620 NT
SW620 TRAM
SW620 DAS
SW620 T+D
DiFi NT
DiFi TRAM
DiFi DAS
DiFi T+D
SW48 NT
SW48 TRAM
SW48 DAS
SW48 T+D
SNU2CB NT
SNU2CB TRAM
SNU2CB DAS
SNU2CB T+D

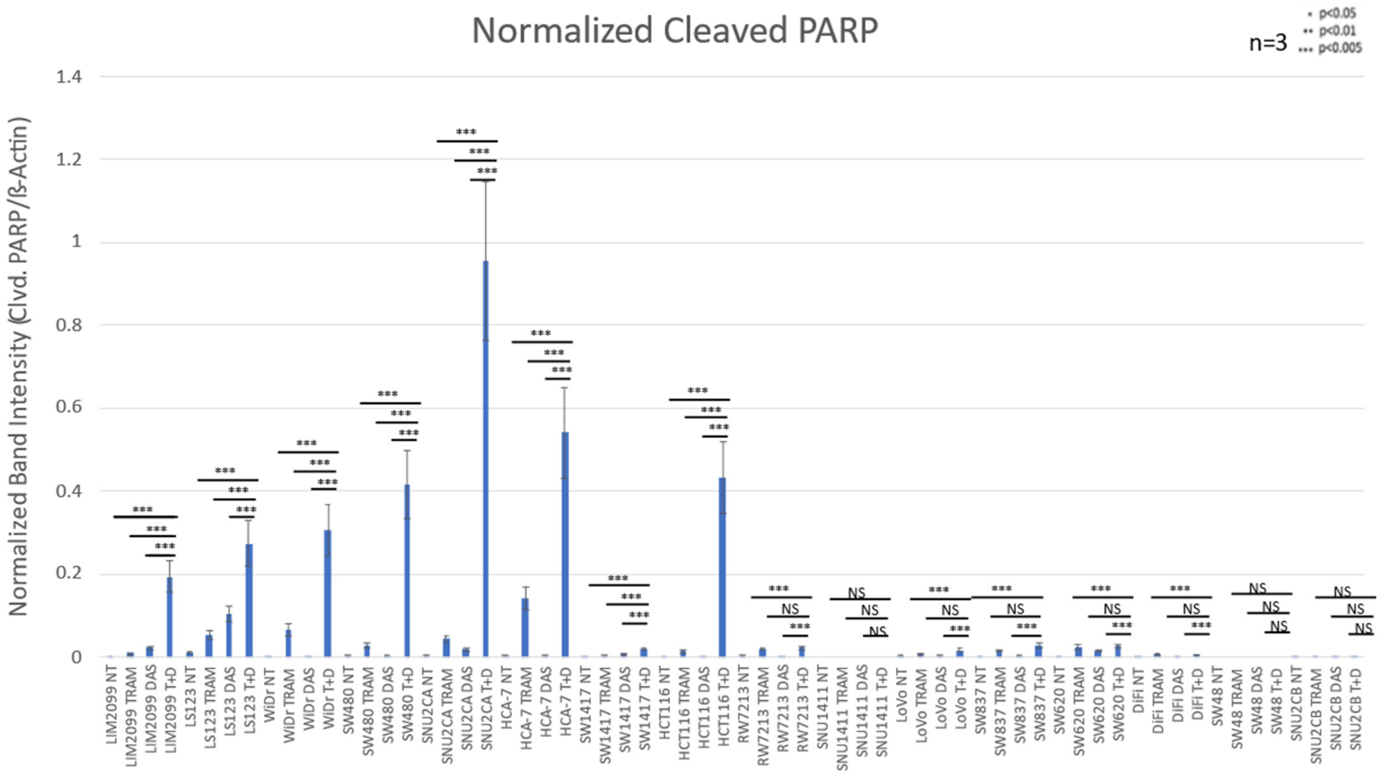

Figure S3. Band Density analysis for western blots of Figure 5.

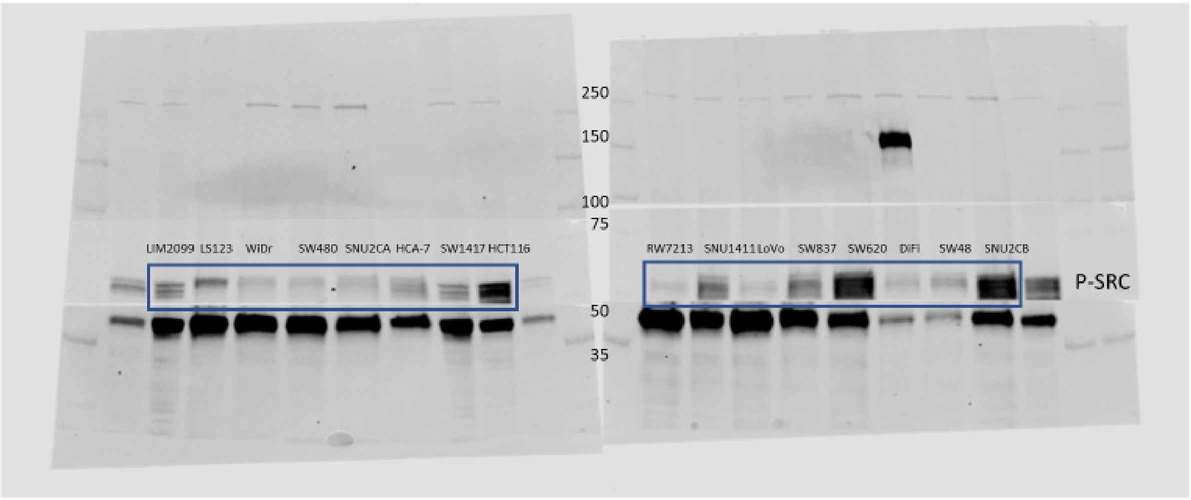

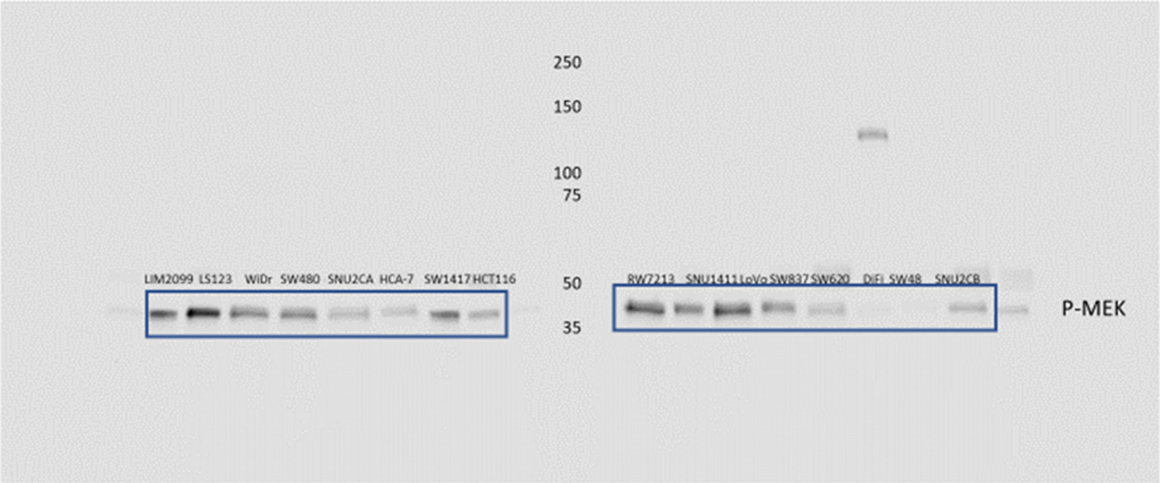
250
150
100
75
50
35
LIM2099 LS123 WiDr SW480 SNU2CA HCA-7 SW1417 HCT116
RW7213 SNU1411 LoVo SW837 SW620 DiFi SW48 SNU2CB
P-MEK

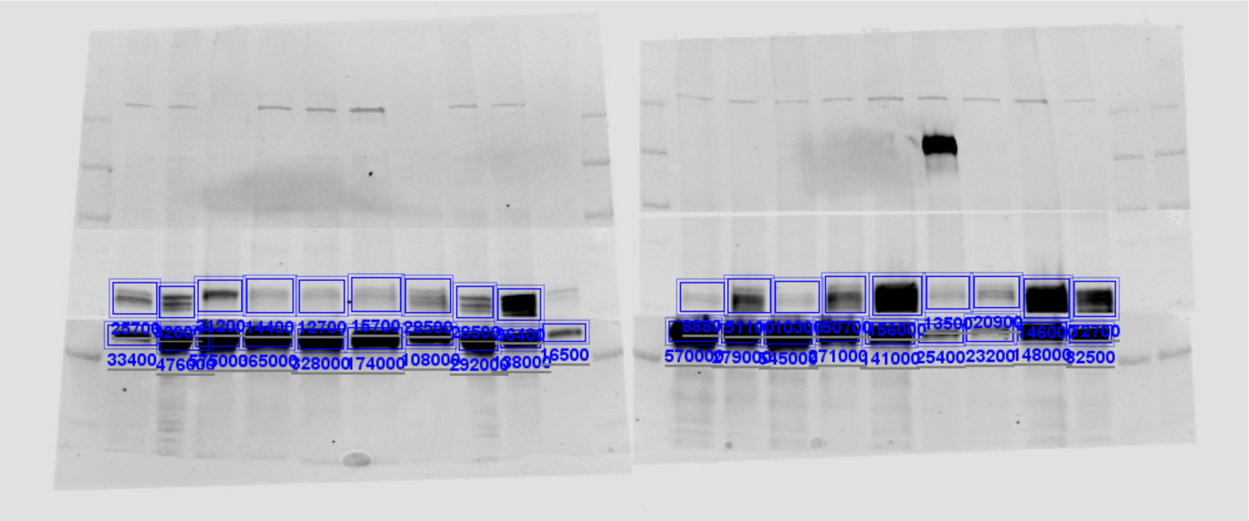

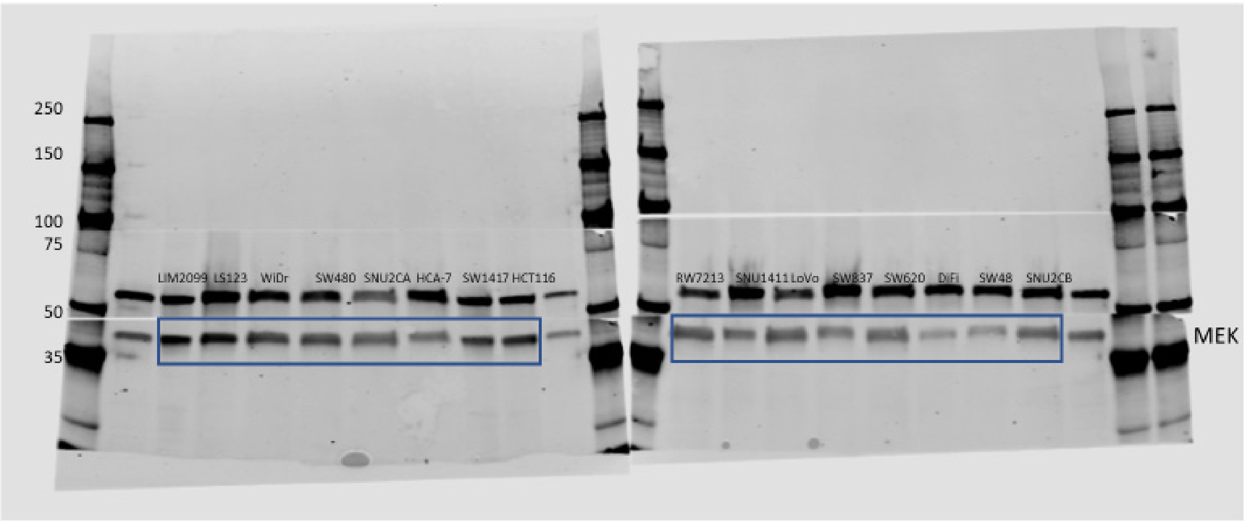
250
150
100
75
50
35
LIM2099 LS123 WiDr SW480 SNU2CA HCA-7 SW1417 HCT116
RW7213 SNU1411 LoVo SW837 SW620 DiFi SW48 SNU2CB
MEK

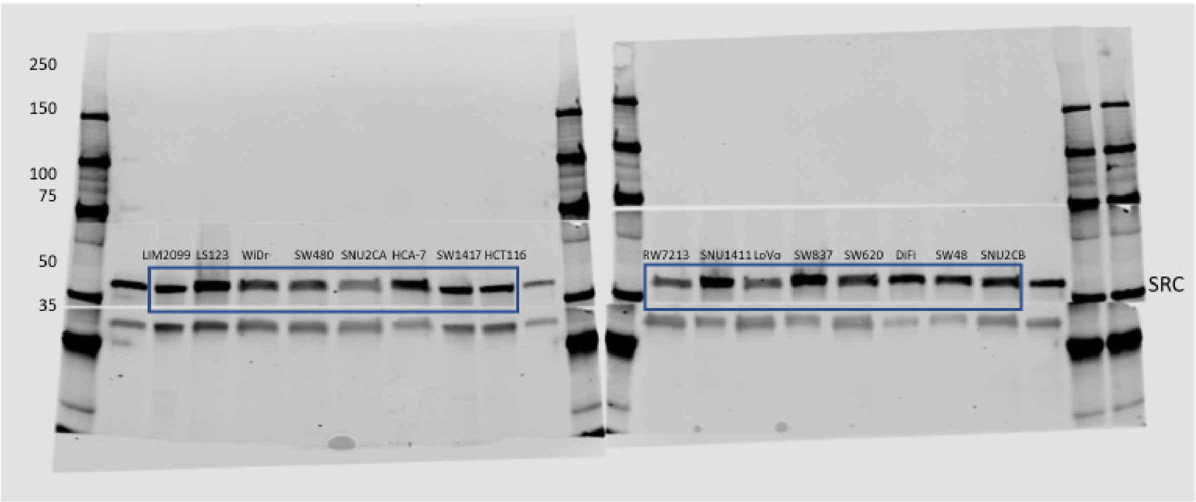
250
150
100
75
50
35
LIM2099
LS123
WiDr
SW480
SNU2CA
HCA-7
SW1417
HCT116
RW7213
SNU1411
LoVo
SW837
SW620
DiFi
SW48
SNU2CB
SRC

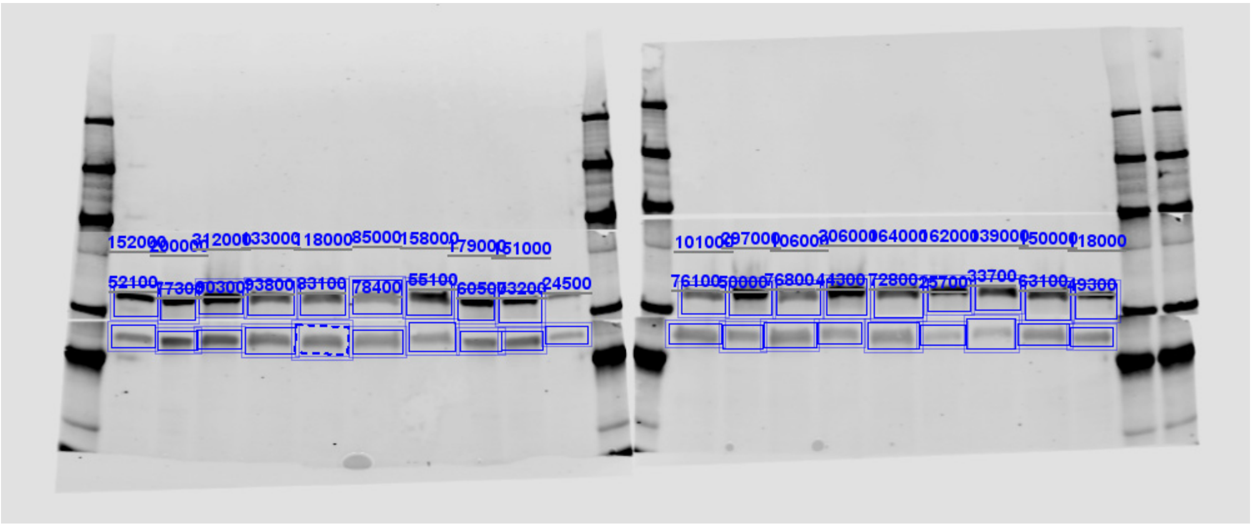
152000
158000
52100
78400
55100
24500
33700
63100

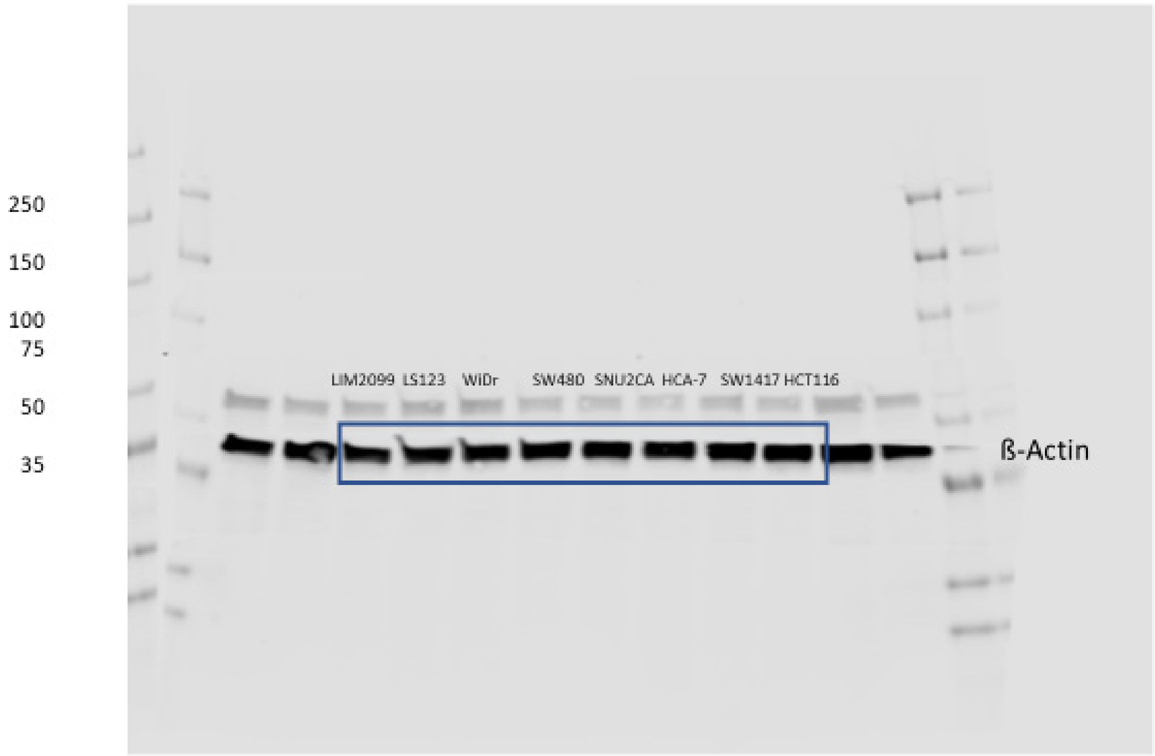
250
150
100
75
50
35
LIM2099
LS123
WiDr
SW480
SNU2CA
HCA-7
SW1417
HCT116
ß-Actin

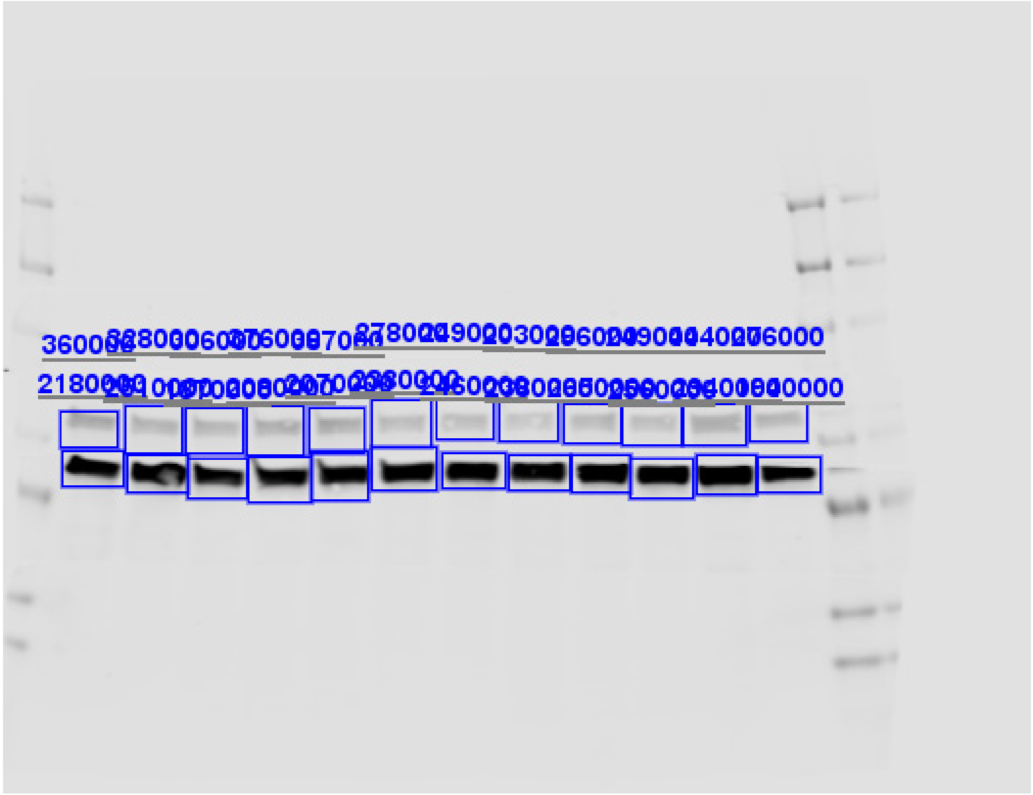

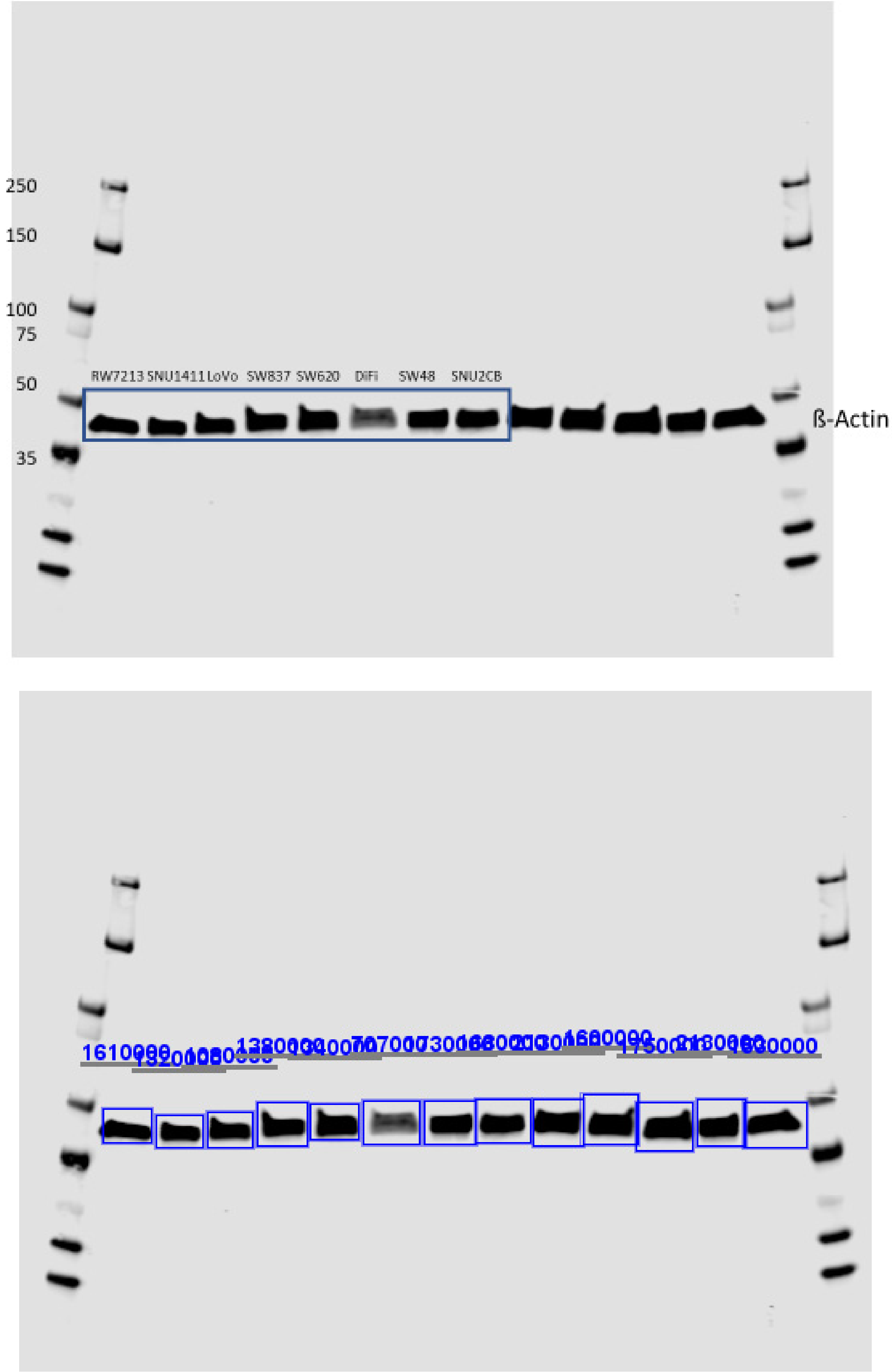

Figure S4. Full western blots of Figure 1.

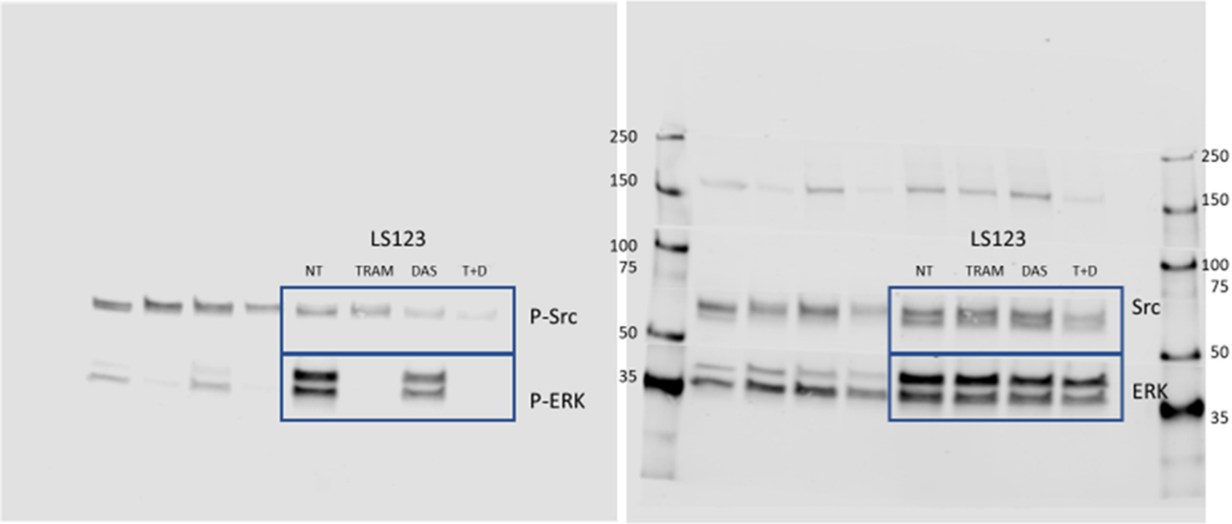
LS123
NT
TRAM
DAS
T+D
P-Src
P-ERK
250
150
100
75
50
35
LS123
NT
TRAM
DAS
T+D
Src
ERK
250
150
100
75
50
35

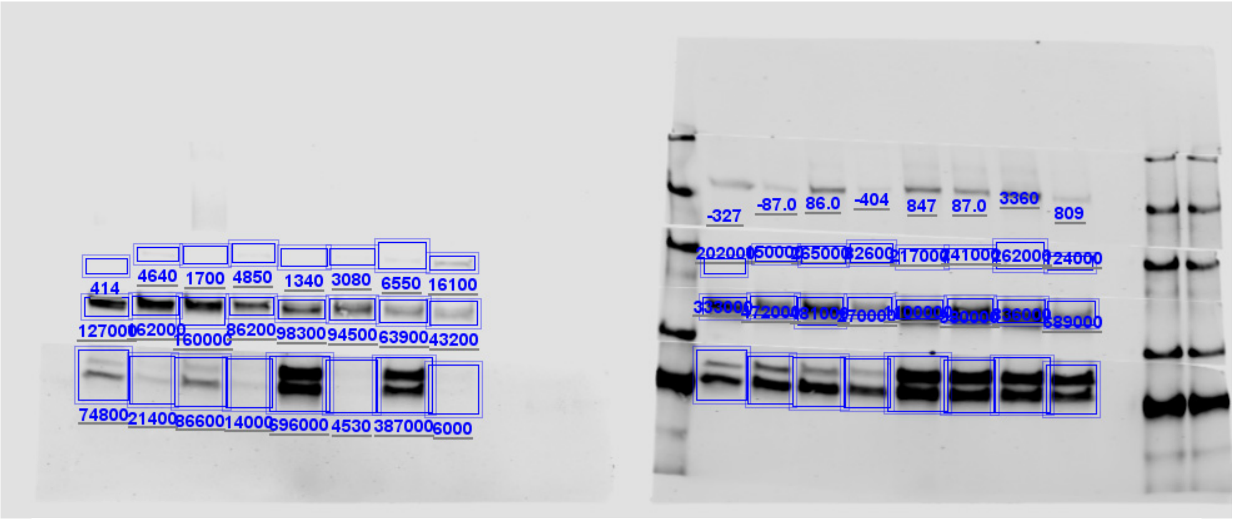
414
4640
1700
4850
1340
3080
6550
16100
86200
98300
94500
63900
43200
74800
14000
696000
4530
387000
6000
-327
-87.0
86.0
-404
847
87.0
3360
809

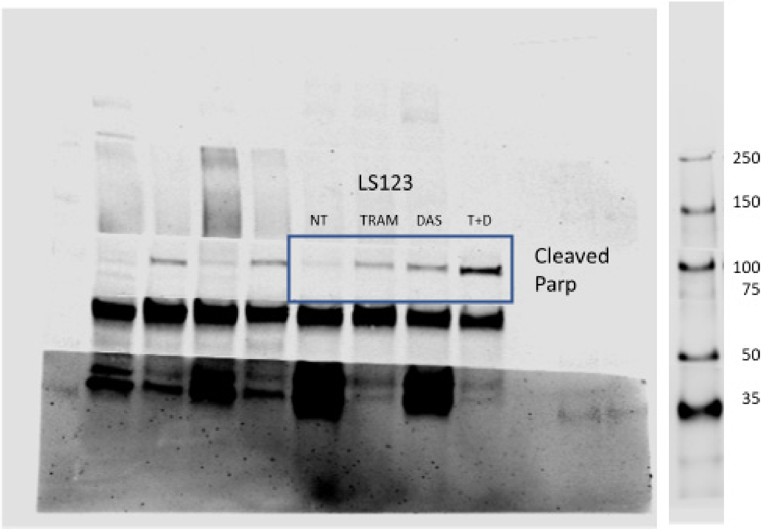
LS123
NT
TRAM
DAS
T+D
Cleaved
Parp
250
150
100
75
50
35

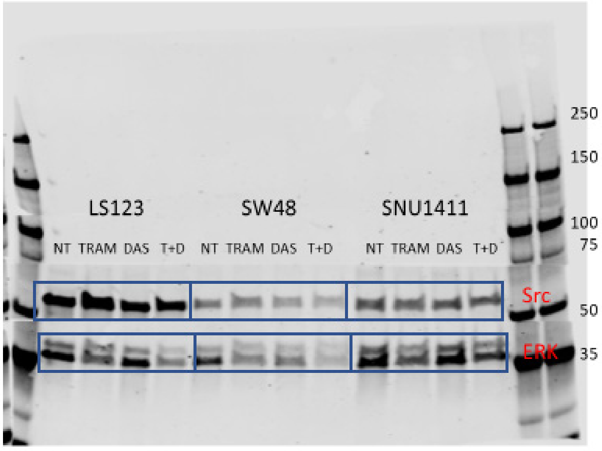
LS123
SW48
SNU1411
NT TRAM DAS T+D NT TRAM DAS T+D NT TRAM DAS T+D
Src
ERK
250
150
100
75
50
35

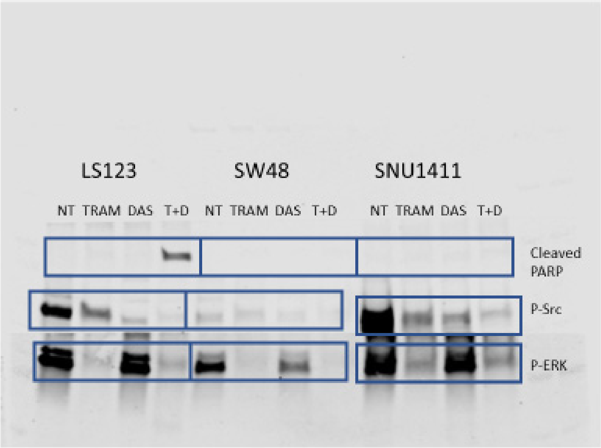
LS123
SW48
SNU1411
NT TRAM DAS T+D NT TRAM DAS T+D NT TRAM DAS T+D
Cleaved PARP
P-Src
P-ERK

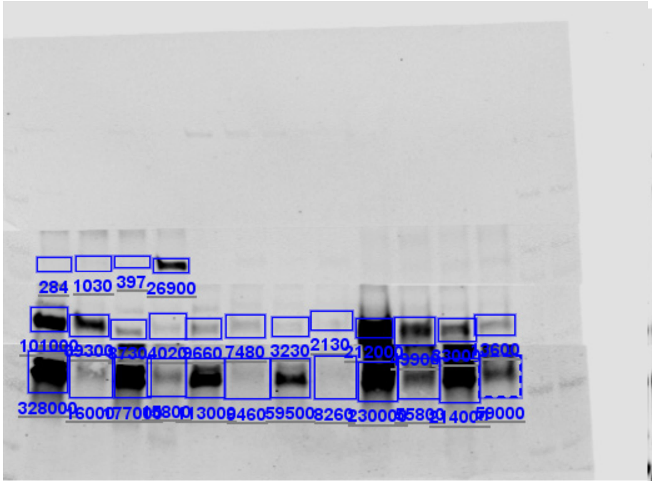
284 1030 397 26900

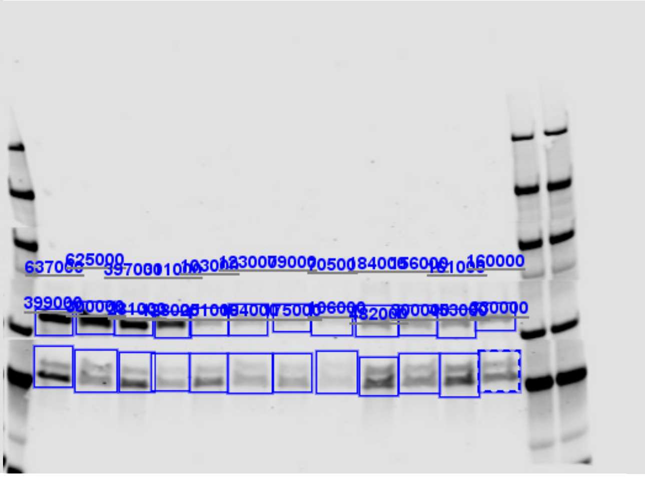

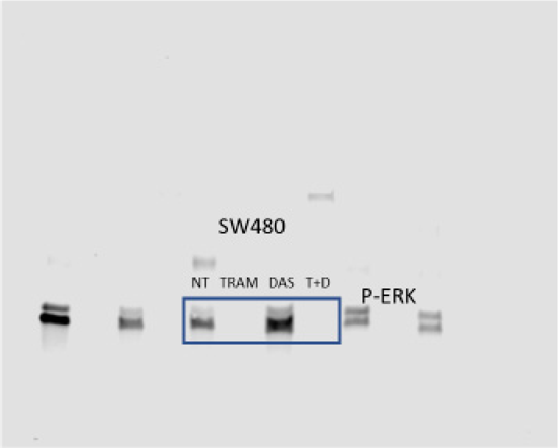
SW480
NT TRAM DAS T+D
P-ERK

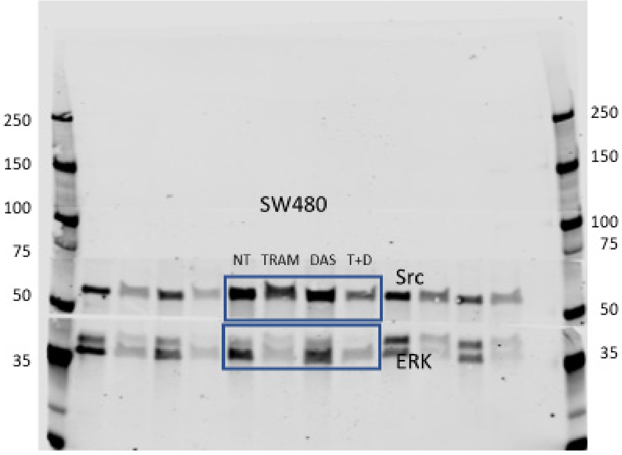
250
150
100
75
50
35
SW480
NT TRAM DAS T+D
Src
ERK
250
150
100
75
50
35

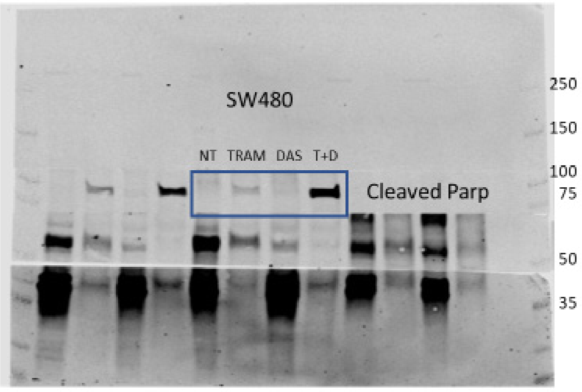
SW480
NT TRAM DAS T+D
Cleaved Parp
250
150
100
75
50
35

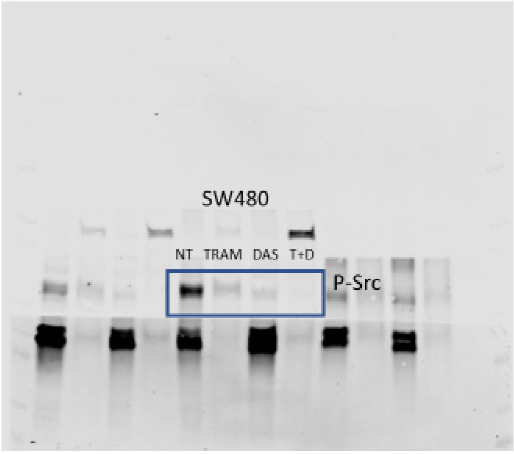
SW480
NT TRAM DAS T+D
P-Src

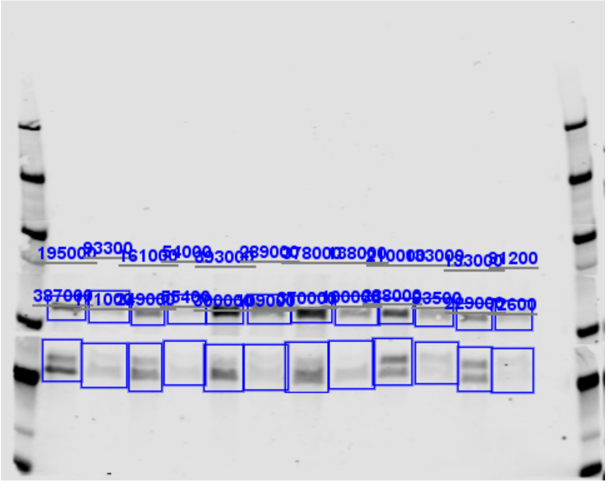

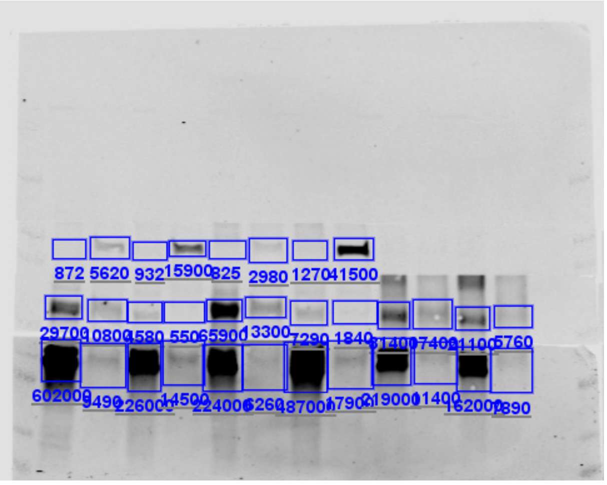

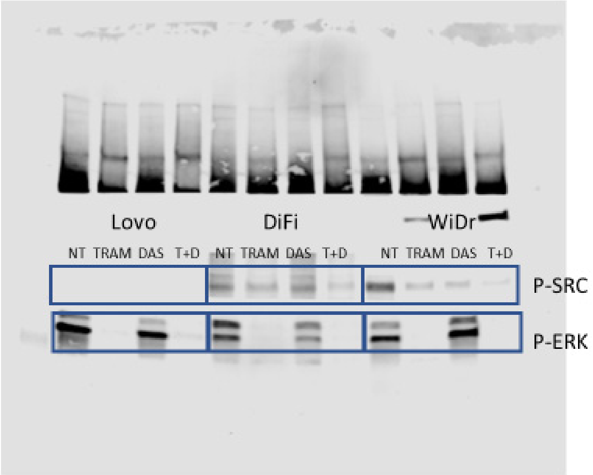
Lovo
DiFi
WiDr
NT TRAM DAS T+D NT TRAM DAS T+D NT TRAM DAS T+D
P-SRC
P-ERK

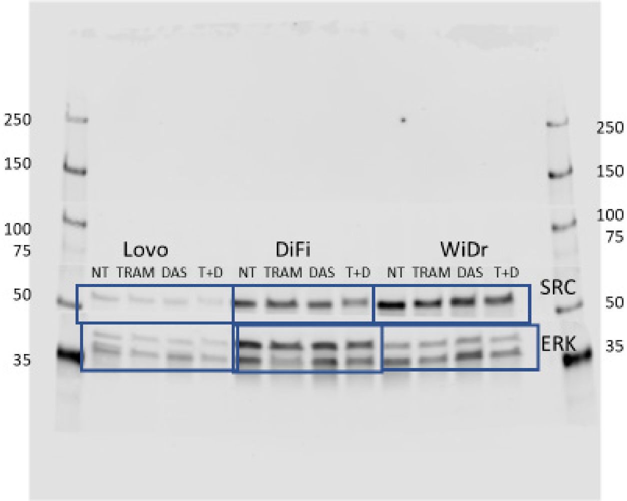
250
150
100
75
50
35
Lovo
DiFi
WiDr
NT TRAM DAS T+D NT TRAM DAS T+D NT TRAM DAS T+D
SRC
ERK

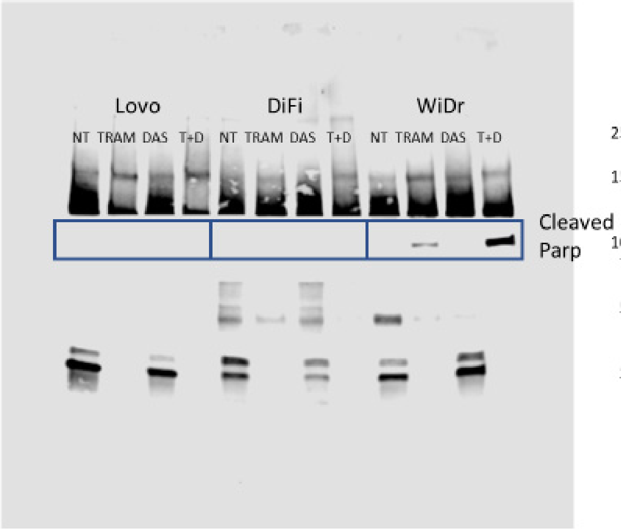
Lovo
DiFi
WiDr
NT TRAM DAS T+D NT TRAM DAS T+D NT TRAM DAS T+D
Cleaved
Parp

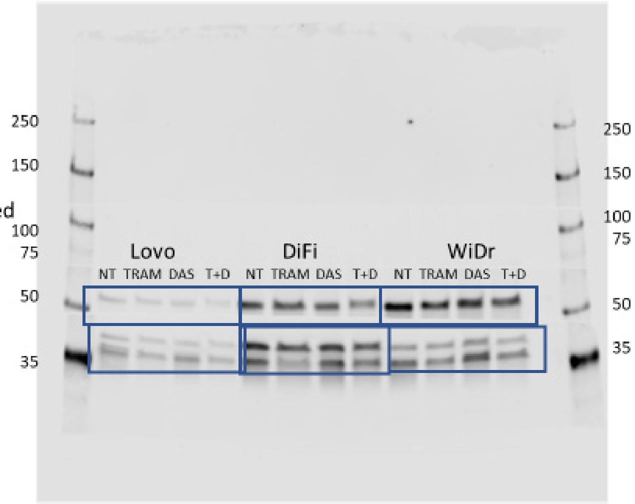
250
150
100
75
50
35
Lovo
DiFi
WiDr
NT TRAM DAS T+D NT TRAM DAS T+D NT TRAM DAS T+D
250
150
100
75
50
35

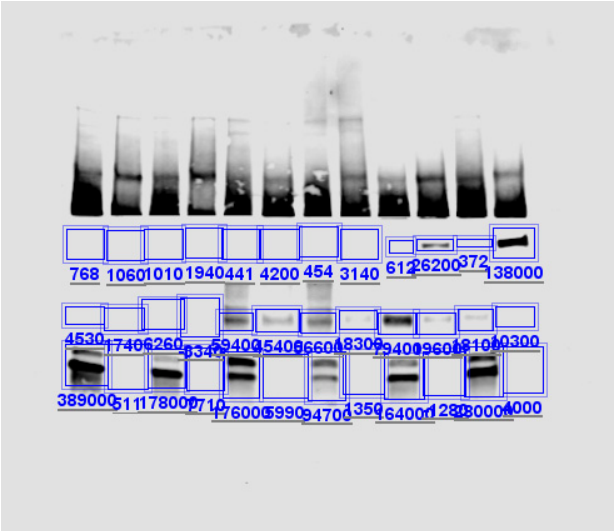

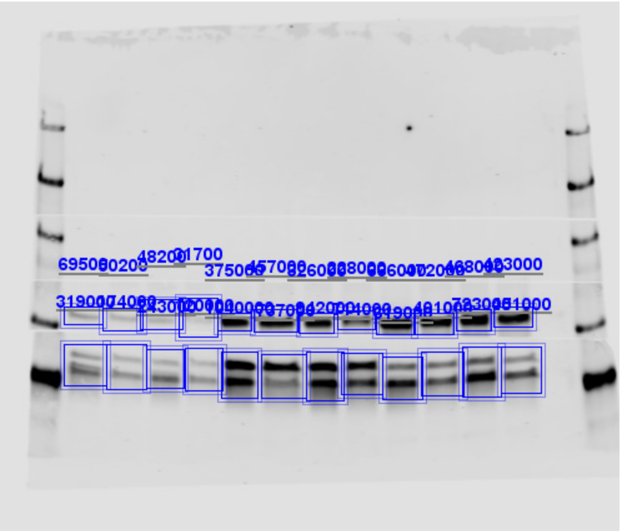

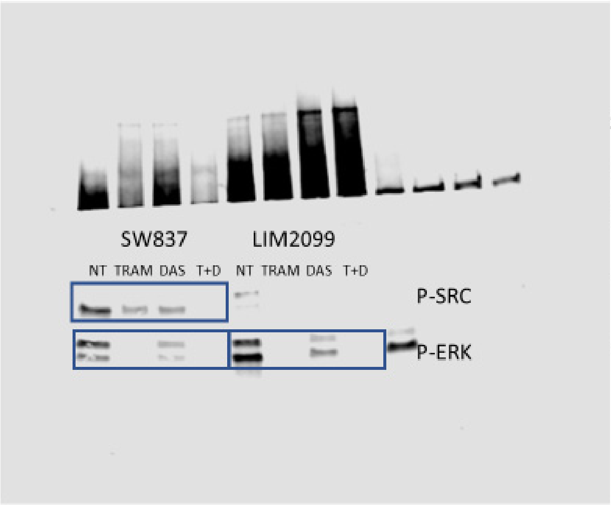
SW837
LIM2099
NT TRAM DAS T+D NT TRAM DAS T+D
P-SRC
P-ERK

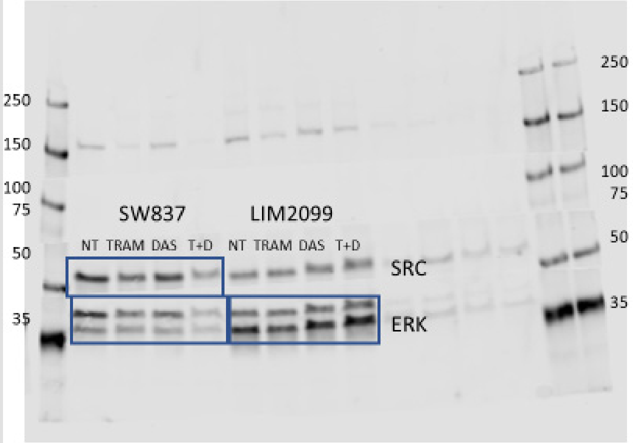
250
150
100
75
50
35
SW837
LIM2099
NT TRAM DAS T+D NT TRAM DAS T+D
SRC
ERK
250
150
100
75
50
35

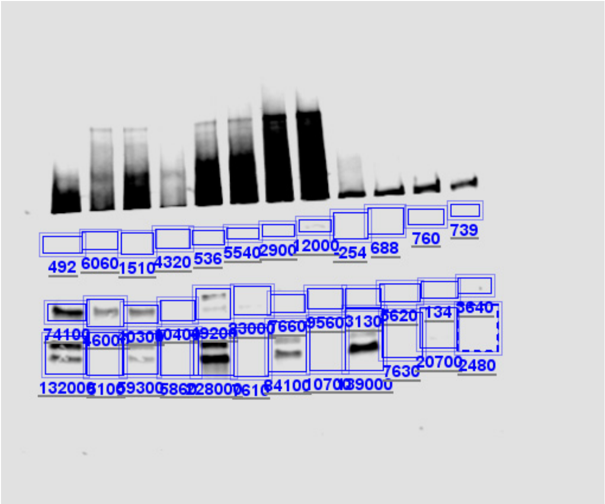

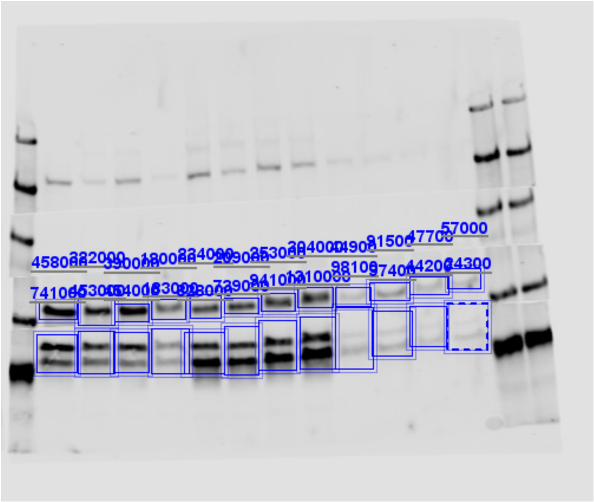

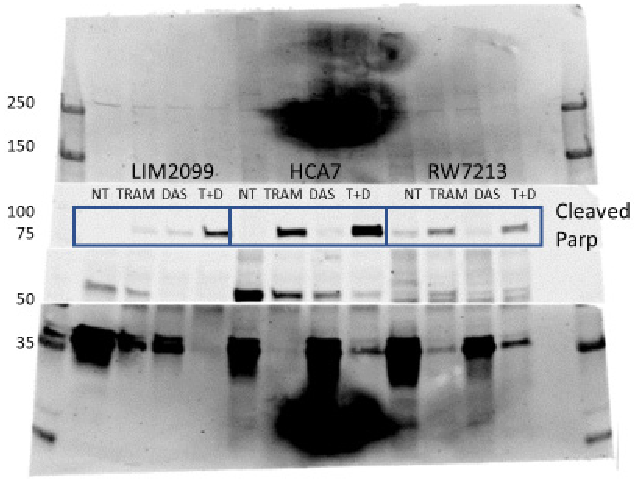
250
150
LIM2099
HCA7
RW7213
NT TRAM DAS T+D
NT TRAM DAS T+D
NT TRAM DAS T+D
100
75
Cleaved
Parp
50
35

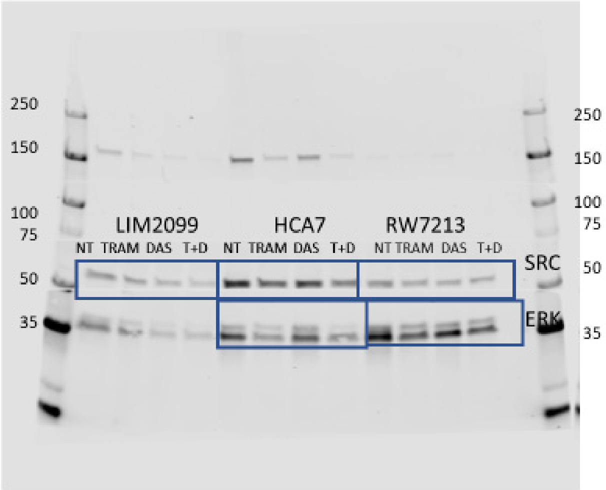
250
150
100
75
LIM2099
HCA7
RW7213
NT TRAM DAS T+D
NT TRAM DAS T+D
NT TRAM DAS T+D
SRC
50
35
ERK

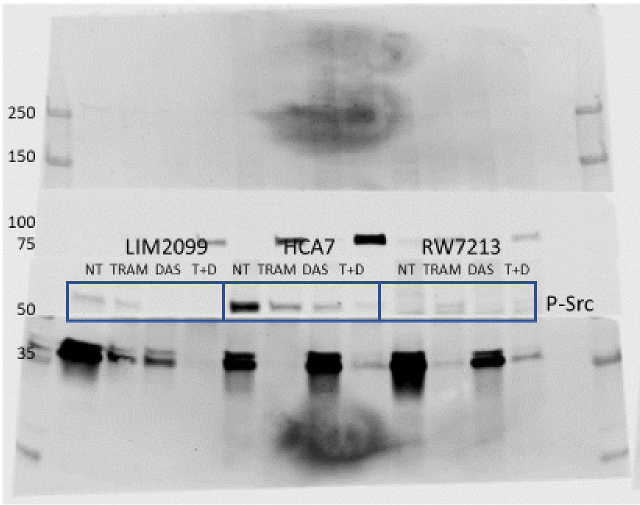
250
150
100
75
LIM2099
HCA7
RW7213
NT TRAM DAS T+D
NT TRAM DAS T+D
NT TRAM DAS T+D
50
P-Src
35

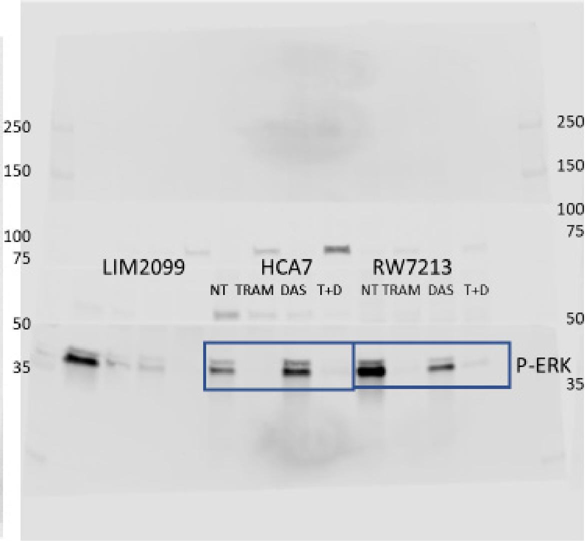
250
150
100
75
LIM2099
HCA7
RW7213
NT TRAM DAS T+D
NT TRAM DAS T+D
50
35
P-ERK

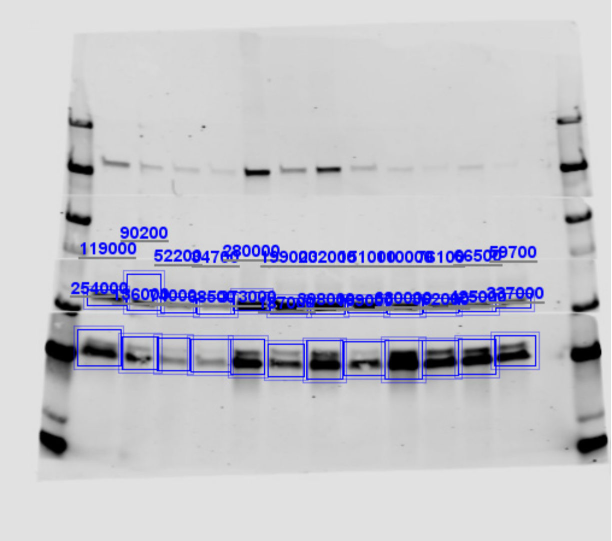

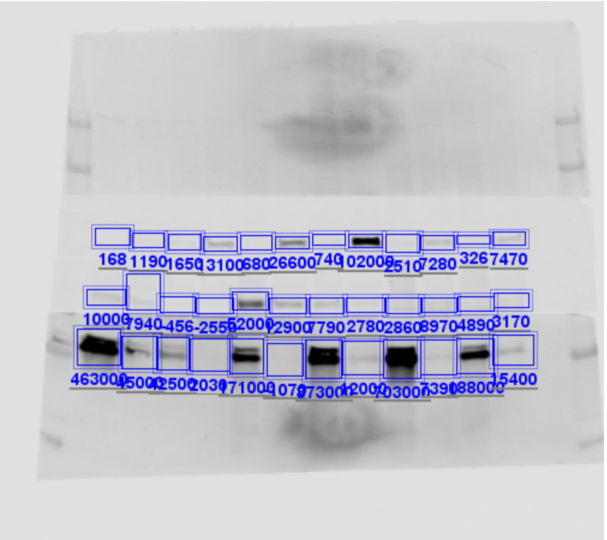

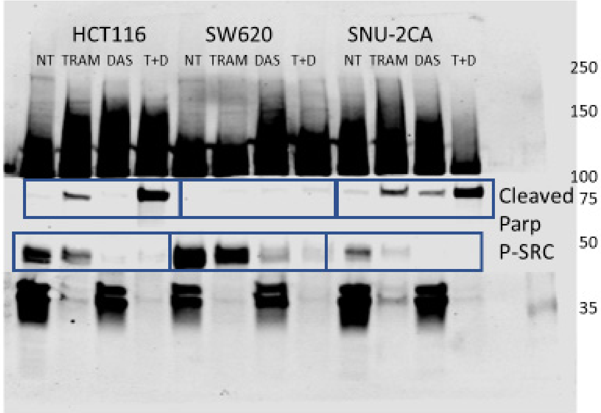
HCT116 SW620 SNU-2CA
NT TRAM DAS T+D NT TRAM DAS T+D NT TRAM DAS T+D
250
150
100
75
Cleaved Parp
50
P-SRC
35

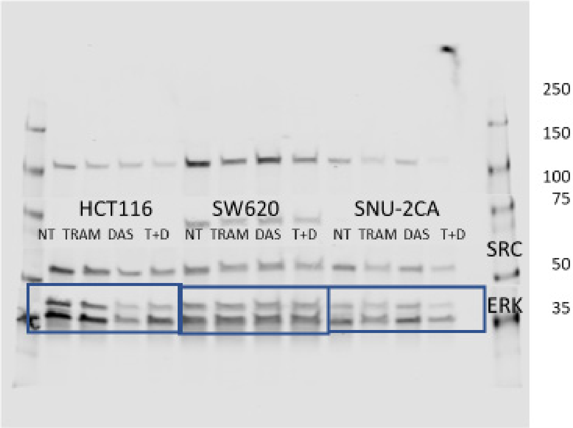
250
150
100
75
HCT116 SW620 SNU-2CA
NT TRAM DAS T+D NT TRAM DAS T+D NT TRAM DAS T+D
SRC
50
ERK
35

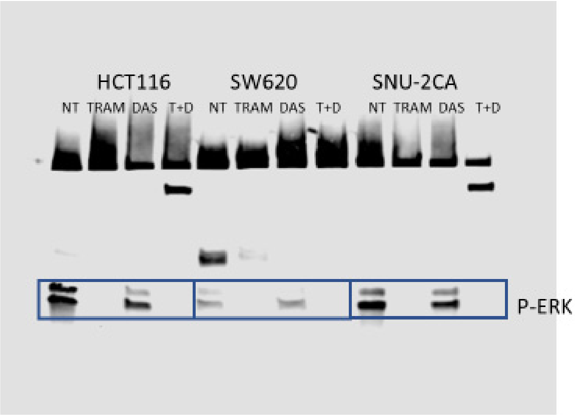
HCT116 SW620 SNU-2CA
NT TRAM DAS T+D NT TRAM DAS T+D NT TRAM DAS T+D
P-ERK

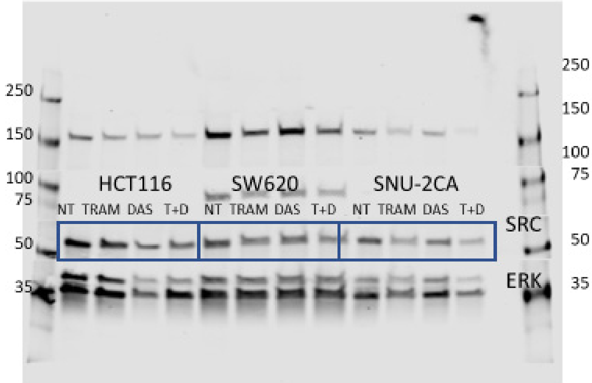
250
150
100
75
50
35
HCT116 SW620 SNU-2CA
NT TRAM DAS T+D NT TRAM DAS T+D NT TRAM DAS T+D
250
150
100
75
SRC
50
ERK
35

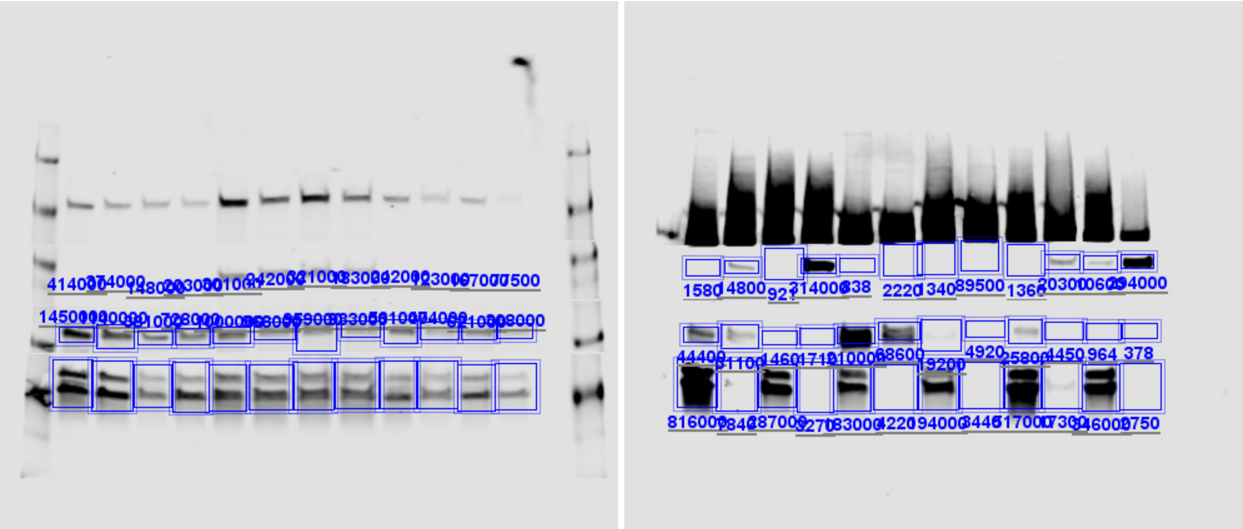

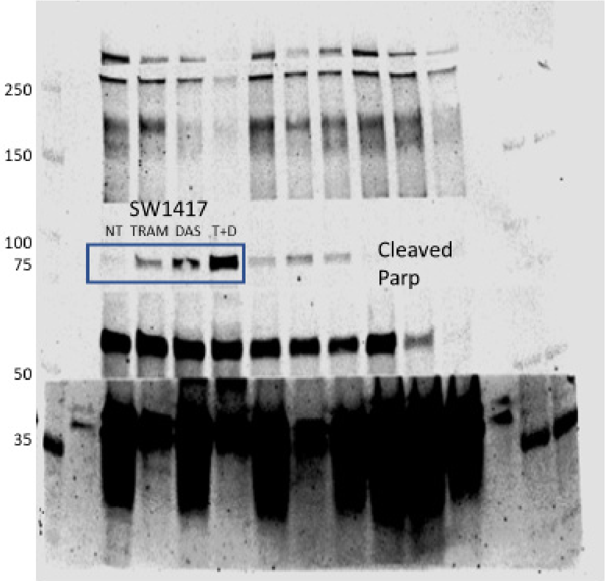
250
150
SW1417
NT TRAM DAS T+D
100
75
Cleaved
Parp
50
35

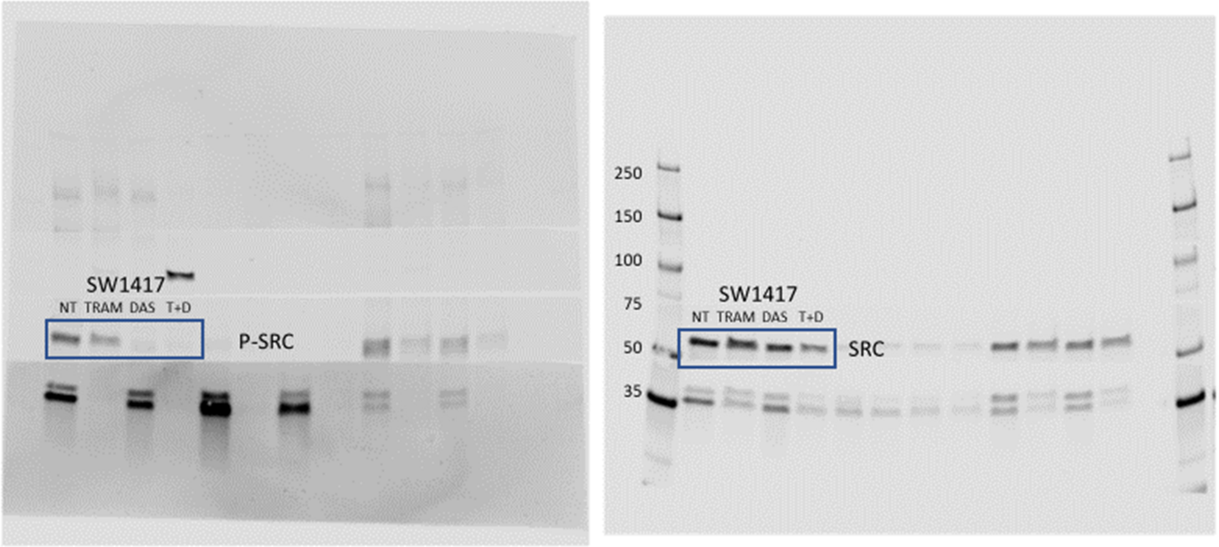
SW1417
NT TRAM DAS T+D
P-SRC
250
150
100
75
SW1417
NT TRAM DAS T+D
50
SRC
35

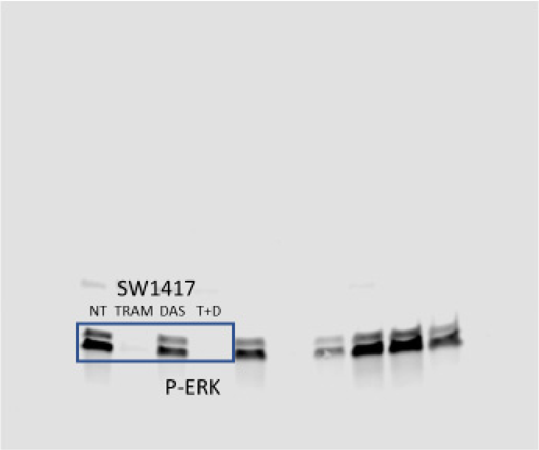
SW1417
NT TRAM DAS T+D
P-ERK

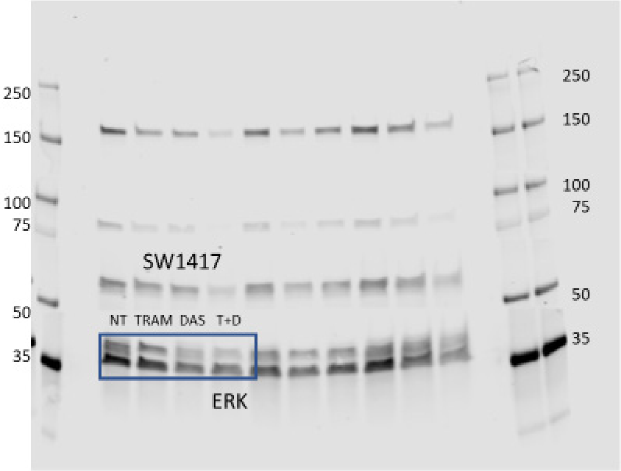
250
150
100
75
50
35
SW1417
NT TRAM DAS T+D
ERK
250
150
100
75
50
35

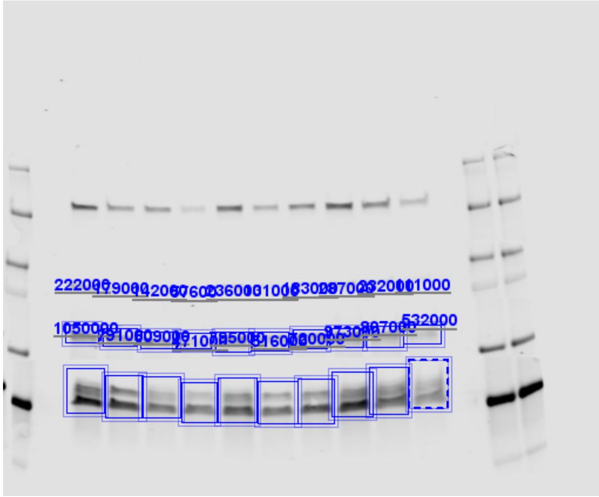

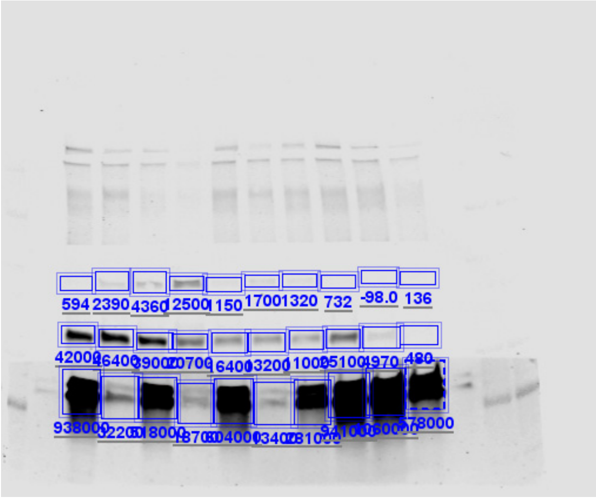

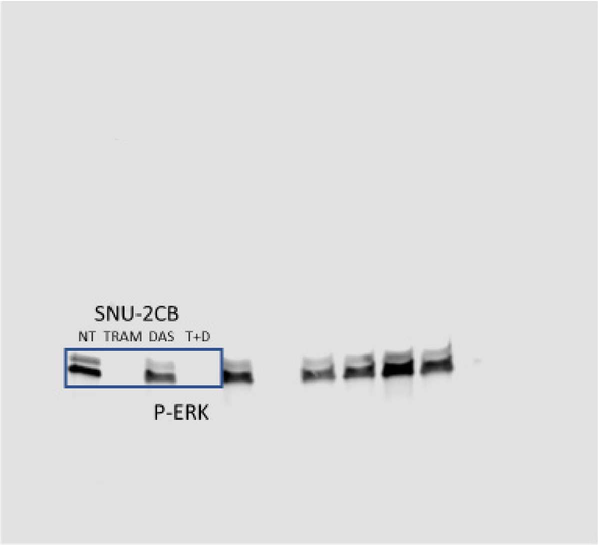
SNU-2CB
NT TRAM DAS T+D
P-ERK

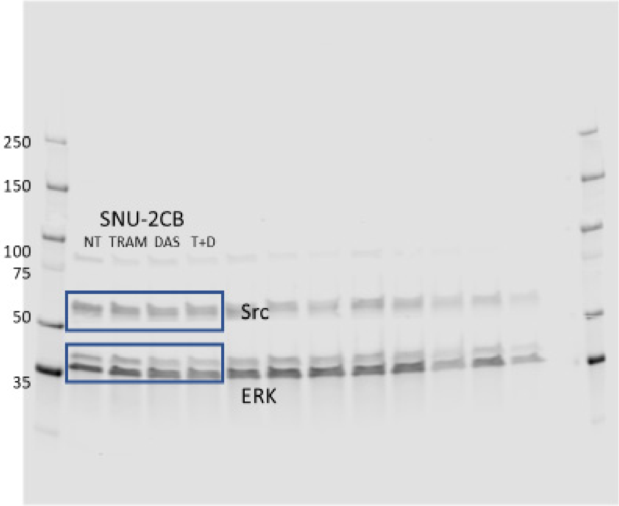
250
150
100
75
50
35
SNU-2CB
NT TRAM DAS T+D
Src
ERK

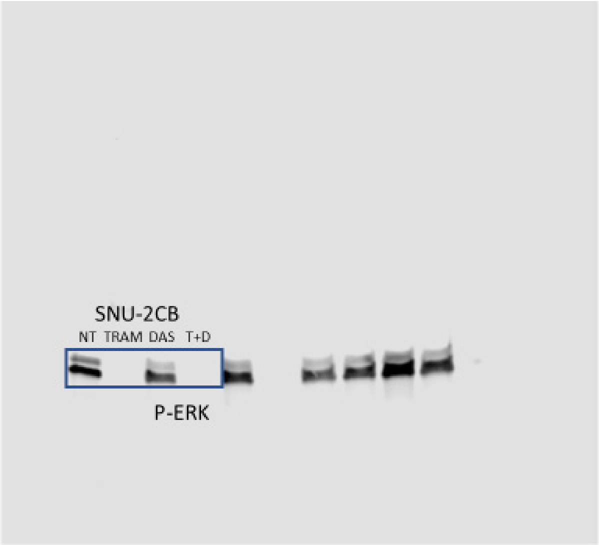
SNU-2CB
NT TRAM DAS T+D
P-ERK

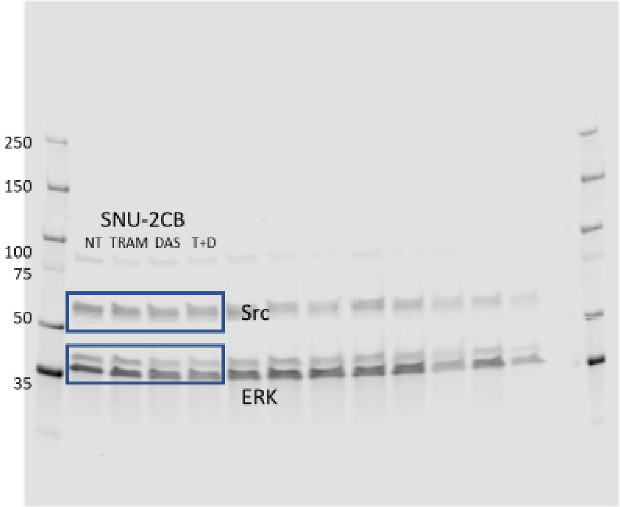
250
150
100
75
50
35
SNU-2CB
NT TRAM DAS T+D
Src
ERK

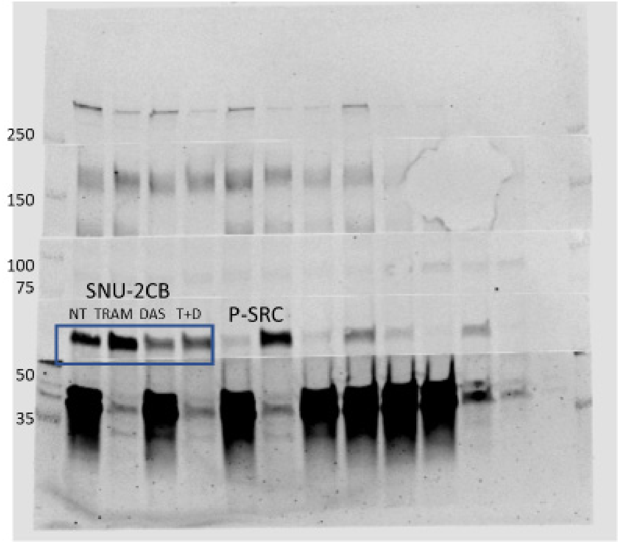
250
150
100
75
50
35
SNU-2CB
NT TRAM DAS T+D
P-SRC

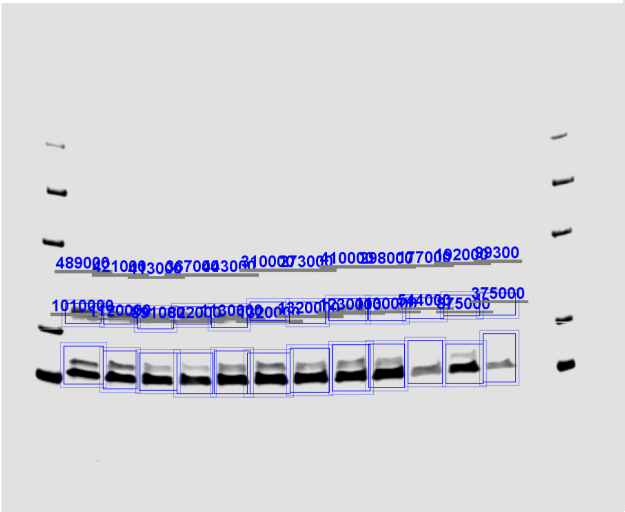

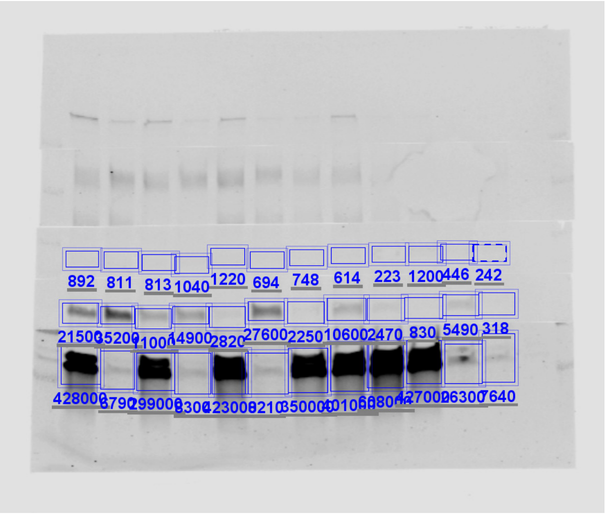
892 811 813 1040 1220 694 748 614 223 1200 446 242
21500 35200 1100 14900 2820 27600 2250 10600 2470 830 5490 318

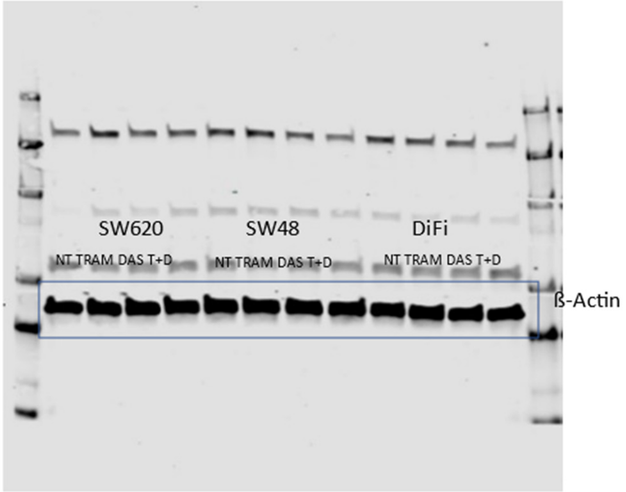
SW620
SW48
DiFi
NT TRAM DAS T+D
NT TRAM DAS T+D
NT TRAM DAS T+D
ß-Actin

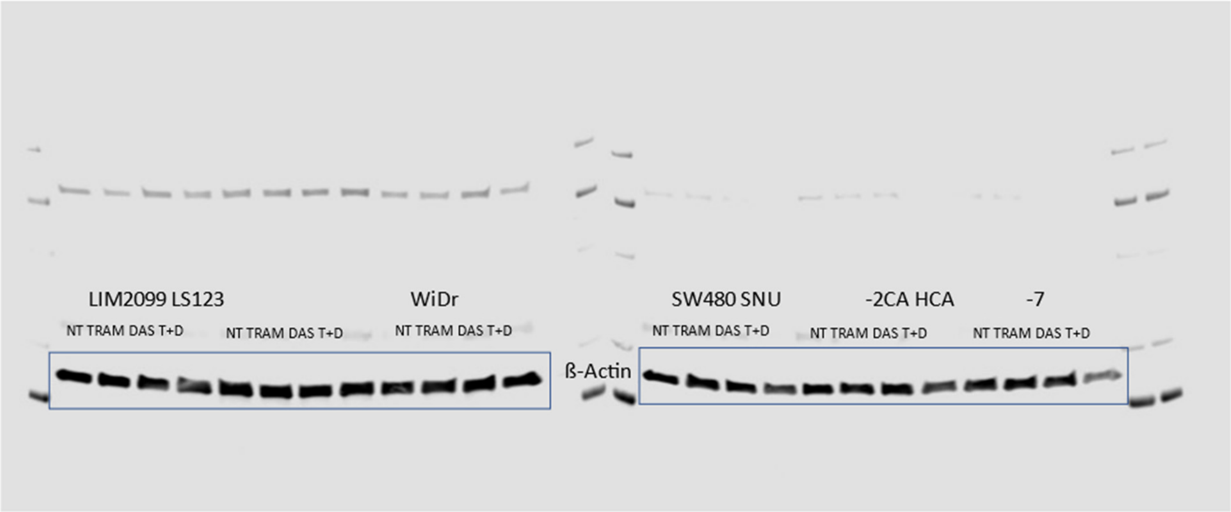
LIM2099 LS123
WiDr
NT TRAM DAS T+D
NT TRAM DAS T+D
NT TRAM DAS T+D
ß-Actin
SW480 SNU
-2CA HCA
-7
NT TRAM DAS T+D
NT TRAM DAS T+D
NT TRAM DAS T+D

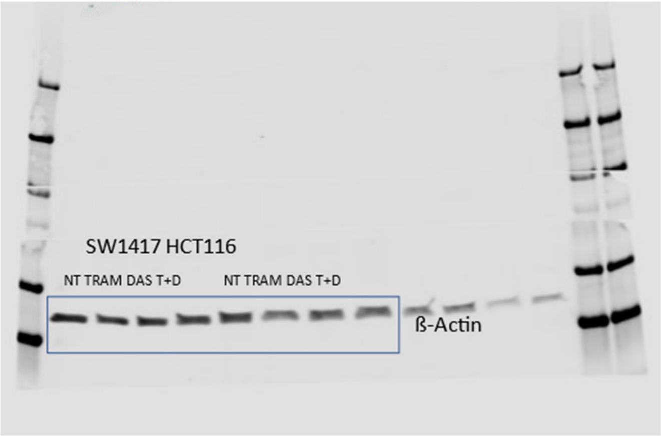
SW1417 HCT116
NT TRAM DAS T+D
NT TRAM DAS T+D
ß-Actin

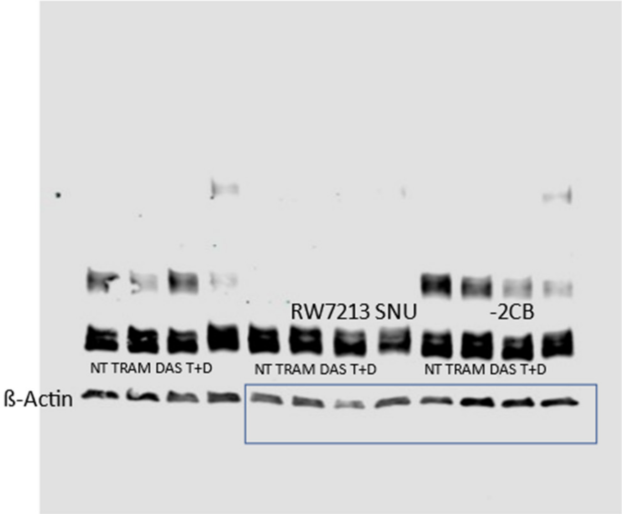
RW7213 SNU
-2CB
NT TRAM DAS T+D
NT TRAM DAS T+D
NT TRAM DAS T+D
ß-Actin

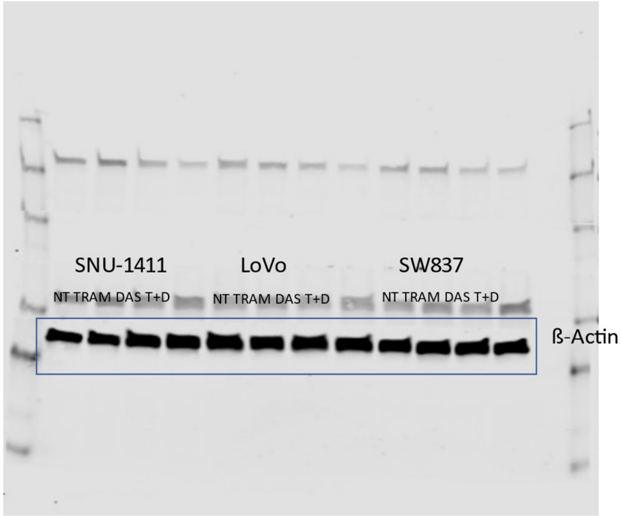
SNU-1411
LoVo
SW837
NT TRAM DAS T+D
NT TRAM DAS T+D
NT TRAM DAS T+D
ß-Actin

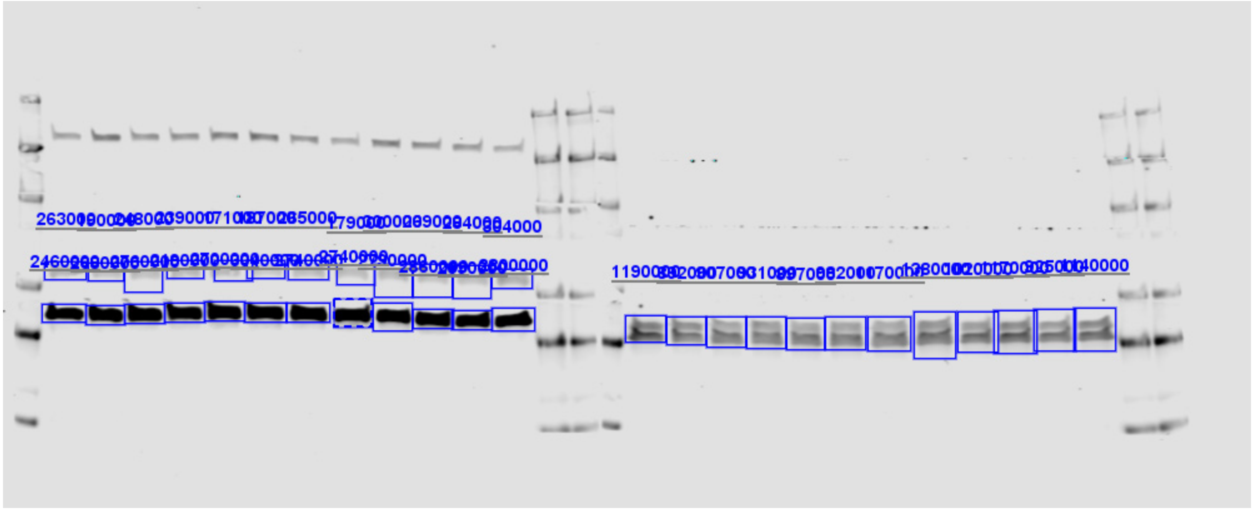

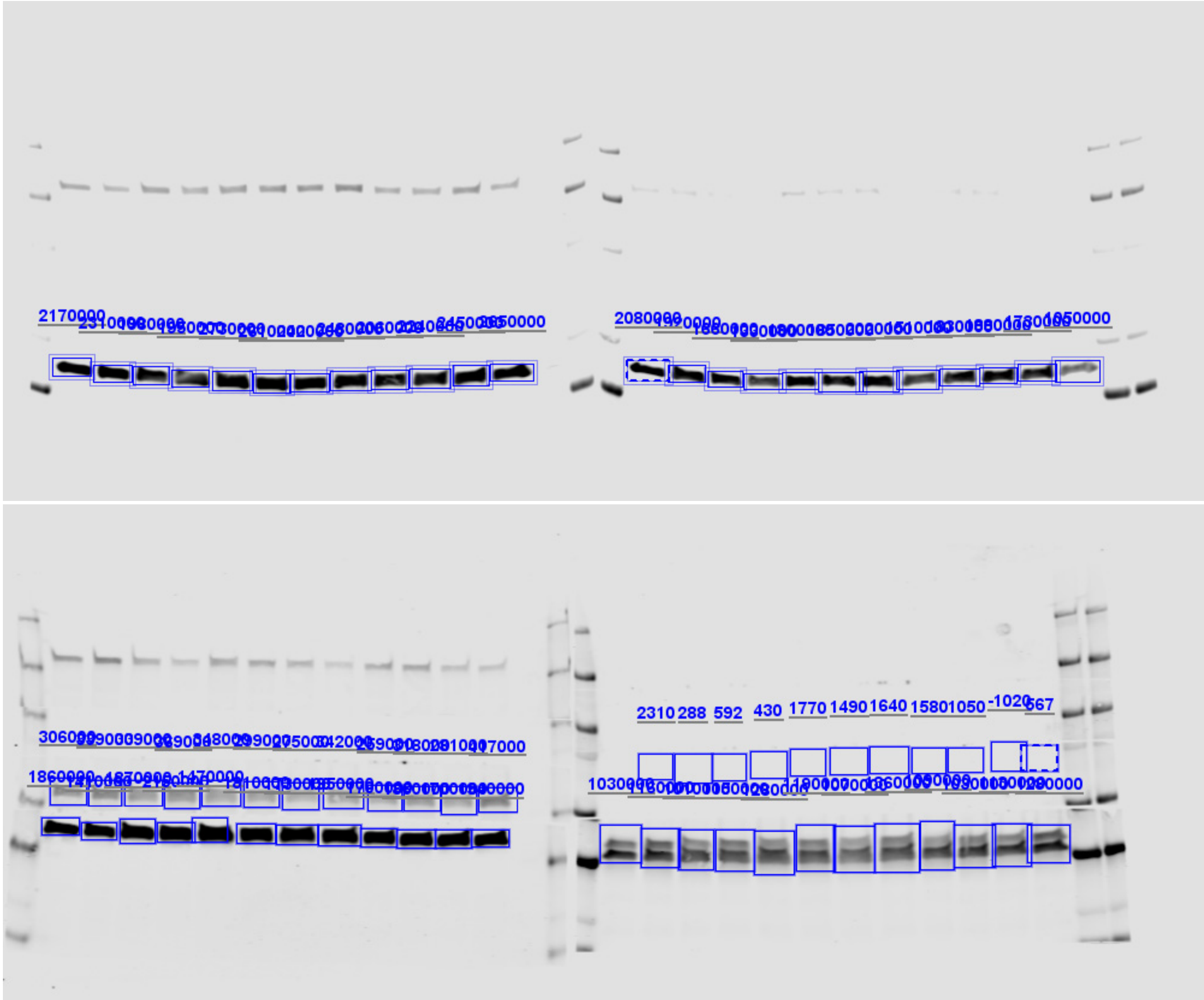

Figure S5. Full western blots of Figure 5.
